# Hemodialysis patients profile at Dow University of Health Sciences, Karachi. Pakistan

**DOI:** 10.12669/pjms.306.5364

**Published:** 2014

**Authors:** Huma Mamun Mahmud, Muneer Siddiqui, Babar Bashir, Syed Farman Ali, Akhter Ali Baloch, Mohd Masroor

**Affiliations:** 1 Dr. Huma Mamun Mahmud, MBBS, FCPS, Consultant Nephrologist, In Charge Hemodialysis Unit, DIMC, Ojha Campus, Dow International Medical College (Ojha Campus), DUHS, Suparco Road, off Main University Road, Gulzar-e-Hijri, Scheme 33, Karachi, Pakistan.; 2 Dr. Muneer Siddiqui, MBBS, FCPS, Dow International Medical College (Ojha Campus), DUHS, Suparco Road, off Main University Road, Gulzar-e-Hijri, Scheme 33, Karachi, Pakistan.; 3 Dr. Babar Bashir, MBBS, FCPS, Dow International Medical College (Ojha Campus), DUHS, Suparco Road, off Main University Road, Gulzar-e-Hijri, Scheme 33, Karachi, Pakistan.; 4 Dr. Syed Farman Ali, Dow International Medical College (Ojha Campus), DUHS, Suparco Road, off Main University Road, Gulzar-e-Hijri, Scheme 33, Karachi, Pakistan.; 5 Dr. Akhter Ali Baloch, Dow International Medical College (Ojha Campus), DUHS, Suparco Road, off Main University Road, Gulzar-e-Hijri, Scheme 33, Karachi, Pakistan.; 6 Dr. Mohd Masroor, Dow International Medical College (Ojha Campus), DUHS, Suparco Road, off Main University Road, Gulzar-e-Hijri, Scheme 33, Karachi, Pakistan.

**Keywords:** ESRF, HBsAg, Anti HCV, DUHS, Hemodialysis

## Abstract

***Objectives: ***To determine the frequency of diseases contributing to End Stage Renal Failure (ESRF) and to determine the frequency of seropositivity for hepatitis B and hepatitis C in our patients.

***Methods: ***This is an observational study of two years duration from January 2012 till December 2013, done at Dow university of Health Sciences. Sample size is 189 by convenient method. Data collection is retrospective. Inclusion criteria includes all patients ever hemodialysed at DIMC with age 14 or above. Exclusion criteria is age below 14. Data maintained and analyzed on SPSS version 16. All categorical data in percentages and numeric data is given in frequencies and mean with Standard deviation.

***Result: ***Total number of patients included in study were 189, Males were 94/189 (49.7%), females were 95/189 (50.3%), Male to female ratio was 0.98: 1.0. Mean age was 51.88+15.2, range was14-86 years. Patients started on Hemodialysis were found to have hypertension in 40.2%, both diabetes and hypertension was present in 42.8%, diabetes alone in 3.1% of patients as likely etiology of renal failure. Seropositivity for HBV was found 4/189(2.1%) and HCV in 31/189(16.4%) at initiation of Hemodialysis.

***Conclusion:*** Hypertension alone is an important disease found in patients with renal failure as likely cause followed by diabetes. Hepatitis C positivity at start of hemodialysis is 16%.

## INTRODUCTION

One of the studies from Pakistan published in an international journal states the origin of ESRF is attributed equally with chronic GN and diabetes mellitus, 33% each, then hypertension 12%, then stone disease 7%, others were less frequently reported causes^1^ While a study published from Nepal reports chronic glomerulonephritis as commonest cause contributing in 40%, followed by Diabetes 17% and hypertension in 13%.^2^

A study from Saudi Arabia shows hypertension 47%, hereditary 23%, and diabetes mellitus in 19% as the reported causes for ESRF.^3^ All these papers are old and considering the changing prevalence of diseases in communities we need to look at current causes of ESRF. Local data on viral serological status on patients who are started on Hemodialysis is lacking.

A recent paper published from India report that Hemodialysis patient’s data is lacking in India and Pakistan because of lack of national registries.^4^ A large majority of patients with ESRD in India seek medical attention late, usually in advanced stages of CKD with uremic complications. Late referral is more frequent in younger patients and those with non-diabetic kidney disease, and is associated with poor socioeconomic status, lack of education and poor outcomes.^5^ With the passage of time more and more elderly patients are started on Hemodialysis which was seen less frequently earlier.^6^^,^^7^

With the help of this study we tried to evaluate cause of renal failure in our patients. With demographic data we would be able to estimate geriatric population frequency started on Hemodialysis in our setup. Frequency of viral hepatitis in our Hemodialysis patients is not well defined. One Saudi paper has described prevalence of anti HCV antibodies among Hemodialysis patients as high as 50%,^3^ while another study found it 57%.^8^ One of the studies done locally in Peshawar involving three Hemodialysis centers found 29.2% of patients positive for hepatitis C antibody.^9^ We tried to look at the frequency of hepatitis B as well as C in our study population and to find out diseases contributing to end stage renal failure by evaluating the available data at time of initiation of Hemodialysis at our campus.

## METHODS

This is an observational retrospective study done from Jan 2012 till December 2013. The sample size was 189 using the confidence level of 95%, calculated through openepi.com by convenient, consecutive method.

***Inclusion criteria:*** all patients ever hemodialyzed at DIMC age at or above 14 years.

***Exclusion criteria:*** age below 14 years.

Data for patients dialyzed at DUHS is maintained on SPSS within Hemodialysis unit. Data entry is done by Hemodialysis in charge and staff on the same day when a patient is registered for Hemodialysis for the first time.

Data included was name, age, gender, HBSAg, Anti HCV, DM and other etiologies of renal failure like hypertension, glomerular disease, stone disease, unknown origin with small kidneys as cause of renal failure and presentation of renal failure whether acute or chronic acute being of less than three month duration, acute on chronic is acute damage on already deranged functions and chronic is of more than three months duration. Hepatitis serology was checked by chemiluminescence Immunoassay.

Data analysis was done on SPSS version 16 for demography, etiology of renal failure and viral serology. All categorical data is given in percentages and numeric data given in frequencies and means with Standard deviation.

## RESULTS

Total number of patients included in the study was 189 which coincides with number of patients registered for Hemodialysis at Dow University Hospital during study period. Males were 94/189 (49.7%), females were 95/189 (50.3%), and Male to female ratio was 0.98: 1.0. Mean age was 51.88±15.2, range was14-86 years. We found 51/189 (26.9%) of patients were above 60 years in age. Frequency of different diseases among study patients is shown in Table-I. Total hypertensive’s were 157/189(83.1%) out of which 81/189(42.8%) were also having diabetes, and 76/189(40.2%) were having hypertension only. Total Diabetics were 87/189 (46%) with 81/189(42.8%) with both diabetes and hypertension and 6/189 (3.17%) were with diabetes only.

Presentation of renal failures was acute in 20/189 (10.6%), acute on chronic renal failure in 40/189(21.1%) and chronic renal failure was in 128/189(67.7%) patients. At the start of Hemodialysis at DUHS seropositivity for HBSAg was seen in 4/189(2.1%), anti HCV positive in 31/189(16.4%), there was one patient who has shown dual positivity. Among 87/189(46%) diabetic patients only one patient had ARF, 20 had acute and chronic renal failure while 66 had CRF. 

## DISCUSSION

End stage renal failure is a serious morbidity with high treatment cost, poor quality of life and high mortality. Our demographic data is showing that patients started on Hemodialysis have no gender discrimination males and females ratio being 0.98:1.0. We also observed that 26.9% of our patients were above 60 year (range 14-86). Hemodialysis in old age patients shows an increasing trend reflecting the overall increased life expectancy,^6^^,^^7^ which also reflected in our patients. We do not follow any particular guidelines but the decision regarding Hemodialysis in old age is taken considering functional status of patients and combined physicians and family wishes. Old patients at Hemodialysis are also with more co morbids and need geriatrics care which is not easily available in our society.

General practitioners in Pakistan are under diagnosing and under treating hypertension,^12^ as around 70% of general practitioners uses incorrect BP cut offs in diagnosing hypertension in elderly and only 34% received treatment.^10^ Despite under diagnosis the prevalence of hypertension in Karachi is reported to be 26 %.^11^ Our results of hypertension being present in 157/189(83.1%) is showing that this is the commonest disease leading to end stage renal failure in our patients. Among them 70(40.2%) were without other known morbidity and 81(42.8%) were with accompanying diabetes mellitus. One of the studies published in 2000 from Pakistan has shown hypertension as a cause of renal failure in only 12% of patients,^1^ from Nepal in 2009 13% were hypertensive’s ^2^ while from Saudi Arabia 47% were having hypertension as cause of renal failure in them.^3^

**Table-I:** Frequency of different diseases among hemodialysis study patients

Hypertension alone	76 (40.2%)
Diabetes alone	6 (3.1%)
Hypertension with diabetes	81 (42.8%)
Glomerular diseases	7 (3.7%)
Obstructive nephropathy	4 (2.1%)
Polycystic kidney disease	1 (0.5%)
Unknown etiology	14 (7.4%)

**Table-II:** Seropositivity of study population

**HBsAg positives**	**Anti HCV positives**	**HBsAg and ANTI HCV both Positive**
4/189	31/189	1/189
(2.1%)	(16.4%)	(0.5%)

The high frequency of hypertension as a cause of renal failure is also indicative of existing high prevalence of hypertension in our society and a strong need for revision of medical under graduates curriculum as well as CME for general practitioners regarding prevention, evaluation and management of hypertension^10^ according to international guidelines. In a local study on diabetics, hypertension was found as a co-morbidity in 48% of patients, while diabetic nephropathy was found in 8%^12^ These results are also similar to our results as 42% of our end stage renal failure patients had both diabetes and hypertension. Reported prevalence of diabetes in Pakistan is 13.1% and impaired fasting in 5.61% .^13^ Another local study published in 2013 is showing increasing trends in hypertension, obesity, smoking which are all associated with increased diabetes risk.^14^

In present study 87/189 patients with end stage renal failure were diabetic. Only six patients had diabetes and rest had hypertension as co morbid or associated factor. These studies and result of our study are indicating a rise in risk factors toward diabetes and also an increase in frequency of diabetes as a cause of renal failure which was reported 33% about one and a half decade ago.^1^

Prevalence of self reported hepatitis C among general population was found to be 0.26% in China and 1.42% in Italy.^15^ A local review of hepatitis B and C prevalence among healthy children was found 1.9-3.6% for hepatitis B and 0.4-4% for hepatitis C.^16^ Prevalence of hepatitis C among general population in Lahore was found to be 4.9% among 4246 samples by HCV PCR assay.^17^

Our study results are showing frequency of hepatitis C among Hemodialysis patients to be 16.4% by chemiluminescene immunoassay. This frequency is reportedly more than three times of what is defined in local study among general population. Frequent blood transfusions though not studied are often required in chronic renal failure patients because of lack of adequate pre dialysis care. This is likely to contribute in this high frequency of hepatitis C at start of Hemodialysis. Also a small number of patients were shifted to our unit after getting Hemodialysis started outside and this may be then falsely indicating this high frequency of HCV positivity in our patients.

Hepatitis B was only seen in 2.1% of our patients likely because of overall reduced prevalence of hepatitis B among general population as a result of inclusion of vaccine as a routine in expanded program on immunization.

A number of patients around us are either unaware of their disease or despite knowing their illnesses are not on regular treatments and follow-ups because of inadequate health facilities or they are not able to comply with medications for poverty. High prevalence of diabetes and hypertension in our society and high frequency of both of these diseases among end stage renal failure demand a strong need to strengthen our health systems regarding management of these chronic illnesses to have preventive role in minimizing end organ damage like renal failure.

***Limitations of the study: ***The sample size is small. Few of the patients who were started on Hemodialysis at DUHS previously had their initial Hemodialysis sessions done outside which might have affected the results of hepatitis serology. Data regarding prior vaccination for HB virus or blood transfusion was also not included which might have affected the results.

## CONCLUSION

Hypertension alone or in combination with diabetes is most common disease leading to end stage renal failure in our patients. Frequency of Hepatitis C among Hemodialysis patients at start of Hemodialysis found considerably high.

## Authors’ contribution:

***Dr Huma Mamun Mahmud: ***Study designing, statistical analysis, accountable for all aspects of work ensuring that questions related to the accuracy or integrity of any part of work are appropriately resolved.

***Dr. Muneer Siddiqui, Dr. Baber Bashir, Dr. Syed Farman Ali, Dr. Akhter Ali Baloch: ***Data collection and manuscript writing.

***Prof. Mohd Masroor: ***Revising it critically and final approval.
